# Effect of intensive care provided by nurse practitioners for postoperative patients: A retrospective observational before-and-after study

**DOI:** 10.1371/journal.pone.0262605

**Published:** 2022-01-21

**Authors:** Kazunao Mori, Yoko Tsukamoto, Satoshi Makino, Takuya Takabayashi, Masahiro Kurosawa, Wataru Ohashi, Masatoshi Okumura, Yoshihito Fujita, Yoshihiro Fujiwara

**Affiliations:** 1 Division of Nursing, Aichi Medical University Hospital, Aichi, Japan; 2 Department of Nursing and Social Sciences, Health Sciences University of Hokkaido, Hokkaido, Japan; 3 Department of Nursing, Aichi Medical University, Aichi, Japan; 4 Division of Biostatistics, Clinical Research Center, Aichi Medical University Hospital, Aichi, Japan; 5 Department of Anesthesiology, Aichi Medical University, Aichi, Japan; Stanford University School of Medicine, UNITED STATES

## Abstract

Nurse practitioners are increasingly now members of intensive care teams in Japan, but no data exist about their effect on the outcomes for critically ill patients. This study aimed to compare the outcomes of postoperative patients on mechanical ventilators before and after the participation of nurse practitioners in intensive care teams. We retrospectively identified 387 patients who underwent postoperative mechanical ventilation at a University Hospital in Japan, using data from medical records from 1 April 2015 to 31 March 2017. We extracted data and compared patients’ length of stay in the intensive care unit and the hospital, mechanical ventilation days, postoperative rehabilitation start date, rehabilitation prescription, intensive care unit and hospital mortality, and intensive care unit readmission. Multiple regression analysis was used to analyze the factors affecting length of stay in the intensive care unit. Patients who received care from nurse practitioners and physicians had significantly shorter stays in intensive care (4.8 ± 4.8 days versus 6.7 ± 10.3 days, *p* < 0.021). Mechanical ventilation days, total length of hospital stay, rehabilitation prescription, mortality in intensive care and hospital, and readmission to intensive care were all similar to those who received care only from physicians. The multiple regression analysis suggests that participation of nurse practitioners in intensive care reduced the length of stay in the unit by 2.6 days (*p* = 0.003). These findings could help to increase use of non-physician healthcare providers in intensive care. Our results demonstrated that it is both effective and safe for nurse practitioners to participate in intensive care teams that provide care for postoperative patients receiving mechanical ventilation.

## Introduction

Recently, nurse practitioners (NPs) have been recognized to have an important role in providing healthcare [1, 2]. Their roles have been increasing in intensive care settings that need interprofessional teams providing collaborative healthcare. In the United States, there are 270,000 certified NPs [3]. The number working in intensive care units (ICUs) has increased since 1990 and this has been associated with reduction in both length of stay in ICU [4, 5] and mortality [6–8].

NP education has been provided at Master’s level in Japan since 2008 [9]. Those who want to become NPs in Japan take a graduate course after gaining five years of clinical experience. After training for two years in graduate school, they must pass the school’s exam to be certified as NPs. There is no national certification scheme. To date, 487 people have been certified as NPs in Japan [10]. NPs are increasingly employed to provide healthcare, but there are no data to demonstrate the effect on patient outcomes.

Our hospital employed two NPs in 2015. They have been involved in managing critically ill patients there since April 2016. To evaluate the safety and efficacy of care provided by NPs, we compared patient outcomes before and after inclusion of NPs on the intensive care team. We considered ICU length of stay, mechanical ventilation days and mortality of patients with and without NP care in the ICU. We hypothesized that ICU length of stay would be decreased with NP care.

## Patients and methods

### Study design

We conducted a retrospective cohort observational study ICU outcomes from 1 April 2015 to 31 March 2017 in Aichi Medical University Hospital. From April 2015 to March 2016, there were no NPs in the ICU team, and all patients were cared for by physicians (physicians group). From April 2016 to March 2017, there were two NPs in the ICU team (NP–physicians group). Patients’ outcomes were compared between the two groups.

### Study population and setting

Postoperative patients receiving mechanical ventilation who stayed in the ICU for > 2 days were eligible for inclusion. The exclusion criteria were two or more admissions to the ICU and no detailed descriptions in the medical records. Limiting to postoperative patients on mechanical ventilators allowed us to more confidently control for case mix and severity of illness across the two comparison groups.

Physicians in the physicians group worked 24-hour shifts, with two or three physicians during the day and two at night. NPs worked 8 hours during the day or 16 hours at night. The staffing for the NP–physicians group was one NP and one or two physicians during the day and one NP and one physician at night. Each group went round the unit twice a day. NPs were onsite most of each day, and available at night. NPs were required to obtain medical histories, and perform physical examinations, adjust intravenous fluids, enteral feeding, and drugs (including catecholamines, sedatives, and antibacterial drugs), interpret clinical laboratory tests and radiographs, change the settings for mechanical ventilation, wean patients off mechanical ventilators, initiate consultation with specialists, remove invasive medical devices, and discuss care issues with patients’ family members. They were not able to insert central venous catheters and chest tubes or prescribe medication. The NPs managed the patients under the supervision of a doctor.

Before the NPs joined the ICU team, ICU length of stay (6.7 days) was longer than the average for the Japanese Intensive Care Patient Database (2.5 days) [11]. To reduce ICU length of stay, the NPs provided mechanical ventilation management and early mobilization. They managed a mechanical ventilator weaning protocol and educated ICU staff to ensure that care quality was consistent with the protocol. This protocol was first introduced in Japan in February 2015, but its importance was not recognized in this ICU. In Japan, there are no respiratory therapists, so mechanical ventilation management was traditionally performed by physicians. Trained registered nurses can change mechanical ventilator settings and provide both ventilator weaning and sedation weaning in Japan, but there were no such nurses in our hospital. The NPs therefore performed the mechanical ventilation management that had previously been provided by physicians, to the same standard. However, they did not make decisions about extubation or discharge from the ICU.

The NPs also encouraged early mobilization to reduce ICU length of stay. This is physical therapy carried out within 2–5 days of admission and has previously been used in our hospital [12]. However, in previous attempts, the physicians, nurses and physiotherapists did not provide collaborative interprofessional care for rehabilitation. The NPs were responsible for deciding whether rehabilitation could be started and performing early mobilization with a physiotherapist and a nurse every morning.

### Data collection

All patient data were collected retrospectively from the electronic medical records. Collected patient characteristics were age, sex, American Society of Anesthesiologists Physical Status (ASA-PS), admission ICU type, Acute Physiology and Chronic Health Evaluation (APACHE) II score [13], Operating time, Anesthesia time, type of surgery, length of stay in ICU and hospital, mechanical ventilation days, rehabilitation prescription, rehabilitation start date, ICU and hospital mortality and ICU readmission. APACHE II score is a severity-of-disease classification system, one of several ICU scoring systems. ASA-PS is a classification of preoperative physical status (PS), which is a system for evaluating the health condition of patients before surgery.

### Study outcomes

The primary outcome was ICU length of stay. The secondary outcomes were mechanical ventilation days, total hospital length of stay, rehabilitation prescription, postoperative rehabilitation start date, ICU and hospital mortality and ICU readmission. To determine whether any other factors were associated with NP care, we investigated the impacts of all factors above on the outcomes.

### Statistical analysis

Descriptive data were presented as number and percentage. Continuous data were described as mean ± standard deviation. Categorical variables were compared using a chi-square test, and continuous data using Student’s *t*-test or the Mann–Whitney *U*-test. A multiple regression analysis was used to evaluate the relationships of age and APACHE II score with the two groups. Differences with *p*-values of < 0.05 were considered statistically significant. We used SPSS version 23.0 software (IBM Corp., Armonk, NY) for all statistical analyses. Sample size calculations used G*Power (version 3.1; Cognitive and Industrial Psychology, Heinrich Heine University, Dusseldorf, Germany). Assuming α = 0.05, β = 0.2 (80% power) and effect size d = 0.5, we needed 128 patients (64 per group).

### Ethical considerations

The study was approved by the ethical committee of our institution (Ethical Committee 18-H099). The collected data were guaranteed anonymity and confidentiality during the study period. Informed consent was waived by the institutional review board because of the retrospective design of the study.

## Results

### Demographic characteristics

**Fig 1:**
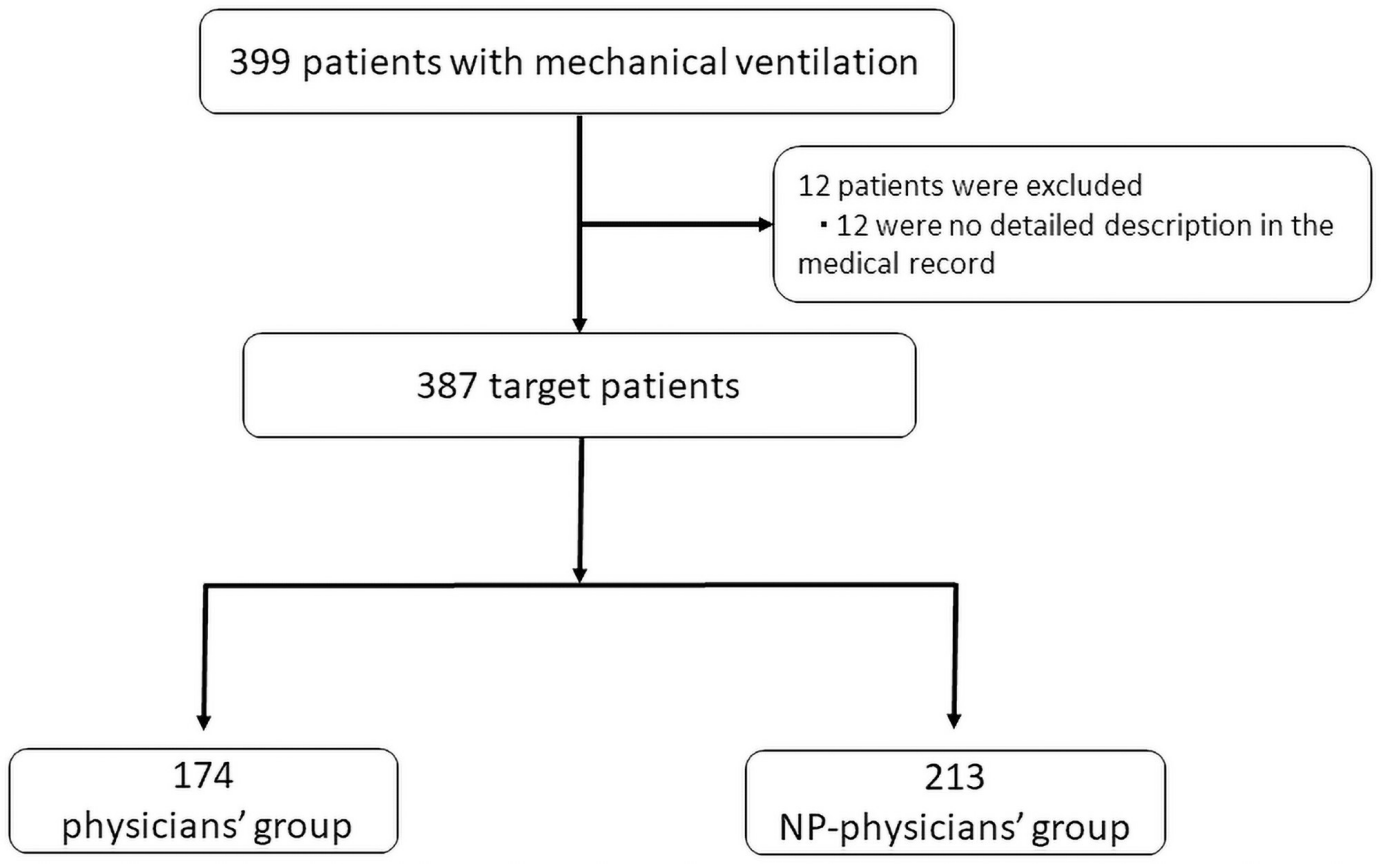
Derivation of the study cohort. All mechanically-ventilated postoperative patient admissions to the physicians group and NP–physicians group were included in the analysis where data were available.

During the two-year study period, 399 postoperative patients requiring mechanical ventilation were admitted to the ICU. In total, 12 were excluded from this study because there was no detailed information in their medical records. After exclusion of these patients, we had data from 387 participants for analysis. In total, 174 were in the physicians group and 213 in the NP–physicians group. The characteristics of the patients at baseline were similar in the two groups, with the exception of emergency surgery and APACHE II score (Fig 1).

The baseline characteristics of the patients in the two groups are summarized in Table 1. Patients in the two groups were similar with respect to age, sex, ASA-PS, operating time and anesthesia time. However, patients in the NP–physicians group tended to have higher APACHE II score and were less likely to have had emergency surgery. There were no significant differences in types of surgery between the two groups, and the biggest group of patients in both groups had had cardiac surgery.

**Table 1:** Characteristics of patients in the ICU.

	Physicians’ group (n = 174)	NP-physicians’ group (n = 213)
Age, mean(SD)	65.6±14.1	64.0±14.8
Male sex–no. (%)	121 (69.5)	138 (64.8)
ASA-PS–no. (%)		
I	10 (5.7)	17 (8.0)
II	72 (41.4)	72 (33.8)
III	88 (50.6)	118 (55.4)
IV	4 (2.3)	5 (2.3)
V	0	1 (0.5)
ICU admission type–no. (%)		
Emergency surgery	33 (19.0)	21 (9.9)
Elective surgery	141 (81.0)	192 (90.1)
APACHE II score, mean(SD) missing, (%)	28.6±5.5	32.0±4.8 1 (0.005)
Operating time (min), mean(SD) missing, (%)	409.6±191.6	387.1±185.4 1 (0.005)
Anesthesia time (min), mean(SD)	506.3±199.0	514.0±812.0
Type of surgery–no. (%)		
Cardiac surgery	93 (53.4)	132 (62.0)
Vascular surgery	1 (0.6)	4 (1.9)
Gastroenterological surgery	35 (20.1)	21 (9.9)
Neurosurgery	29 (16.7)	26 (12.2)
Chest surgery	3 (1.7)	3 (1.4)
Urology surgery	3 (1.7)	1 (0.5)
Head and neck surgery	5 (2.9)	16 (7.5)
Transplant surgery	1 (0.6)	1 (0.5)
Orthopedic surgery	0	1 (0.5)
Plastic surgery	2 (1.1)	3 (1.4)
Obstetrics and gynecology surgery	2 (1.1)	3 (1.4)
Dermatological surgery	0	1 (0.5)
Oral and Maxillofacial surgery	0	1 (0.5)

There were no significant differences between the groups with regard to any baseline characteristic except emergency surgery (P = 0.012), APACHE II score (p<0.001).

ASA-PS: American Society of Anesthesiologists Physical Status.

APACHE II score: Acute Physiology and Chronic Health Evaluation II score.

### Patient outcomes

In the univariate analysis, patients in the NP–physicians group had shorter ICU length of stay (4.8 ± 4.8 days versus 6.7 ± 10.3 days, *p* < 0.021), and earlier postoperative rehabilitation start date (1.88 ± 1.85 days versus 2.93 ± 2.8 days, *p* < 0.001). Mechanical ventilation days, total length of stay in hospital, rehabilitation prescription, ICU and hospital mortality, and ICU readmission were similar in both groups (Table 2).

**Table 2:** Impact of NP participation in the ICU team.

Patient outcomes	Physicians’ group (n = 174)	NP-physicians’ group (n = 213)	P value
ICU length of stay, mean(SD) missing, %	6.7±10.3	4.8 (±4.8) 2 (0.009)	0.021
mechanical ventilation days, mean(SD) missing, %	3.2±6.0	2.2±6.8 3 (0.01)	0.105
total hospital length of stay, mean(SD) missing, %	40.5±58.2	37.1±36.6 1 (0.005)	0.483
Rehabilitation prescription–no. (%)	136 (78.2)	164 (77.0)	0.808
Postoperative rehabilitation start date(SD)	2.93±2.8	1.88±1.85	<0.001
ICU mortality–no. (%)	3 (1.7)	4 (1.8)	1.0
Hospital mortality–no. (%)	6 (3.4)	4 (1.8)	0.355
ICU readmission–no. (%)	7 (4)	10 (4.6)	0.808

ICU: Intensive care unit

Patients in the NP–physicians group had significantly shorter ICU length of stay.

The results of the multiple regression analysis are shown in Table 3. In the model, age and postoperative rehabilitation start date had no impact on ICU length of stay. However, having emergency surgery, APACHE II score and being in the NP–physicians group were independently associated with ICU length of stay. Emergency surgery was significantly associated with an increase in ICU length of stay of 3.5 days. A one-point decrease in APACHE II score was significantly associated with a reduction of 0.2 days in ICU length of stay. Being in the NP–physicians group was significantly associated with a 2.6-day reduction in ICU length of stay.

**Table 3:** Multiple regression analysis for ICU length of stay.

Variables	B	SE	β	T value	P value
age	−0.041	0.029	−0.073	−1.400	0.162
Emergency surgery	3.548	1.150	0.157	3.006	0.003
APACHEII score	0.203	0.080	0.140	2.538	0.012
Postoperative rehabilitation start date	-0.125	0.116	-2.341	-1.074	0.283
NP-physician group	−2.562	0.845	−0.162	−3.031	0.003

APACHE II score: Acute Physiology and Chronic Health Evaluation II score

Shorter ICU length of stay was associated with APACHE score and being in the NP–physicians group.

## Discussion

In this retrospective study of postoperative patients on mechanical ventilators, we found that NP participation in the ICU team was associated with reduced ICU length of stay, but it is unclear whether the association was directly causal. Total length of hospital stay, ICU and hospital mortality, and ICU readmission rates were unaffected.

These findings are important for hospitals considering adding NPs to ICU teams. We found that ICU length of stay was 2.6 days lower in the NP–physicians group. In the multivariate analysis, ICU length of stay was associated with having emergency surgery, APACHE II score rating and the addition of NPs to the ICU team. Emergency surgery was associated with increased ICU length of stay, and a decrease in APACHE II score and the participation of NPs in the ICU team with reduced time in ICU. In previous studies, ICU length of stay was prolonged in patients who were more severely ill at admission [14]. In particular, ASA-PS score of IV and needing emergency surgery were related to ICU length of stay [15]. In this study, there was no significant difference with ASA-PS, but it is possible that the ICU stay was longer because there were more patients receiving emergency surgery in the physicians group than in the NP–physicians group. ICU mortality and ICU readmission rate were similar, suggesting that there were no safety implications from NPs caring for postoperative patients needing mechanical ventilation. The NPs took over the mechanical ventilation management previously performed by physicians, and this had no negative implications for care quality.

NPs could have better impact on postoperative patients on mechanical ventilators because they reduced the ICU length of stay. NPs provided mechanical ventilation management, promoted early mobilization, and educated ICU staff in patient care management and quality assurance of postoperative patients receiving mechanical ventilation. Mechanical ventilator was managed by observing subtle changes in the patient’s condition, such as changes in vital signs trends and signs of clinical deterioration. In addition, promoting early mobilization and educating ICU staff may have been able to facilitate implementation and adherence to mechanical ventilation weaning protocols. It was particularly important that NPs promoted interprofessional team collaborative care. NPs frequently discussed with RNs, physiotherapists and pharmacists to provide best care for patients. As a result of NPs intervention, there was no significant difference in mechanical ventilator days, but the postoperative rehabilitation start date was 1 day earlier in patients in the NP–physicians group. In previous studies, early mobilization reduced mechanical ventilator days and ICU length of stay [16], but we found no relationship with ICU length of stay. Patient safety and the presence of a team are barriers to early mobilization [12, 16]. The role of NPs in managing patient care and performing early mobilization with nurses and physiotherapists may have promoted early mobilization. The number of intensivists in the ICU team before and after the addition of NPs did not change, and neither did the overall medical care system in the ICU. However, there are many factors that affect ICU length of stay. Therefore, it is impossible to say categorically that the addition of NPs to the ICU team caused the reduction in ICU length of stay. However, we might speculate that part of the effect of NPs is through improved communication. There is evidence from elsewhere that NPs can improve collaborative care provided by interprofessional teams [17] and this may have happened here. NPs play an important role in coordinating interprofessional team collaborative care and providing support to ICU staff at night [18]. However, we did not measure communication among the ICU team in this study. Further research is therefore needed to explore the mechanisms behind this finding.

In summary, our study found that NP participation in the ICU team was associated with reduced ICU length of stay. NPs provided mechanical ventilation management and promoted early mobilization, but neither of these factors showed a statistically significant relationship with length of stay. In the future, research is needed to examine whether the link is directly causal. We speculate that NPs may be improving outcomes of postoperative patients on mechanical ventilators by improving communication between teams, and providing training and support for nurses, but this would also need further research to examine more closely.

This study is significant because it is the first report about outcomes associated with NP employment in ICUs in Japan. However, it had some limitations. First, decisions about extubation and discharge from the ICU are subjective and may vary between intensivists and anesthesiologists. However, NPs did not take these decisions, and there was no change in the physicians making either decision during the study period, so we believe that the impact on the study was minimal. Second, our study was conducted in the ICU of an academic university hospital and was supervised by intensivists and anesthesiologists. There are no respiratory therapists in Japan and the approach to mechanical ventilator management may not be global. The results of this study therefore cannot be generalized to other ICUs, open ICUs or pediatric ICUs. Third, this study was a retrospective review, rather than a prospective randomized controlled trial. It would not have been possible to carry out a randomized controlled trial because we could not divide the patients into groups with or without NP care in our setting. The numbers of patients therefore could not be unified in the two groups. Fourth, the study does not suggest that NPs can replace physicians. This study was supervised by intensivists and anesthesiologists and involved all eligible patients during the study period. Our finding did not clearly indicated NPs independent impact on postoperative patients.

## Conclusions

We found that patients treated by both NPs and physicians had shorter ICU length of stay and that there was a significant association between NP participation in the ICU team and ICU length of stay. Although the independent effect of NP on postoperative patients is not clear, we have identified that NP participation in the ICU team provide effective care for postoperative patients receiving mechanical ventilation. Further, research is needed to establish whether the link is directly causal.

## Supporting information

S1 Table (XLSX)
